# The Fastest Flights in Nature: High-Speed Spore Discharge Mechanisms among Fungi

**DOI:** 10.1371/journal.pone.0003237

**Published:** 2008-09-17

**Authors:** Levi Yafetto, Loran Carroll, Yunluan Cui, Diana J. Davis, Mark W. F. Fischer, Andrew C. Henterly, Jordan D. Kessler, Hayley A. Kilroy, Jacob B. Shidler, Jessica L. Stolze-Rybczynski, Zachary Sugawara, Nicholas P. Money

**Affiliations:** 1 Department of Botany, Miami University, Oxford, Ohio, United States of America; 2 Department of Chemistry and Physical Science, College of Mount St. Joseph, Cincinnati, Ohio, United States of America; Dartmouth College, United States of America

## Abstract

**Background:**

A variety of spore discharge processes have evolved among the fungi. Those with the longest ranges are powered by hydrostatic pressure and include “squirt guns” that are most common in the Ascomycota and Zygomycota. In these fungi, fluid-filled stalks that support single spores or spore-filled sporangia, or cells called asci that contain multiple spores, are pressurized by osmosis. Because spores are discharged at such high speeds, most of the information on launch processes from previous studies has been inferred from mathematical models and is subject to a number of errors.

**Methodology/Principal Findings:**

In this study, we have used ultra-high-speed video cameras running at maximum frame rates of 250,000 fps to analyze the entire launch process in four species of fungi that grow on the dung of herbivores. For the first time we have direct measurements of launch speeds and empirical estimates of acceleration in these fungi. Launch speeds ranged from 2 to 25 m s^−1^ and corresponding accelerations of 20,000 to 180,000 *g* propelled spores over distances of up to 2.5 meters. In addition, quantitative spectroscopic methods were used to identify the organic and inorganic osmolytes responsible for generating the turgor pressures that drive spore discharge.

**Conclusions/Significance:**

The new video data allowed us to test different models for the effect of viscous drag and identify errors in the previous approaches to modeling spore motion. The spectroscopic data show that high speed spore discharge mechanisms in fungi are powered by the same levels of turgor pressure that are characteristic of fungal hyphae and do not require any special mechanisms of osmolyte accumulation.

## Introduction

The nature of spore release mechanisms among fungi has been investigated since the eighteenth century [1], and contemporary analysis of these extraordinary processes has implications for the fields of plant disease control, terrestrial ecology, indoor air quality, atmospheric sciences, veterinary medicine, and biomimetics [2]–[6]. Mechanisms include a catapult energized by surface tension that launches mushroom spores, the explosive eversion of a pressurized membrane in the artillery fungus, and the discharge of squirt guns pressurized by osmosis [7]. Squirt gun mechanisms are responsible for launching spores at the highest speeds and are most common in the Ascomycota, including lichenized species, but have also evolved among the Zygomycota [8]. In the so-called “coprophilous” fungi in both phyla, specialized for growth on herbivore dung, these squirt gun mechanisms propel spores over distances of many centimeters or even meters onto fresh vegetation where they may be consumed by their host animals. The range of these mechanisms necessitates very high launch speeds to counteract the otherwise overwhelming influence of viscous drag on the flight of microscopic projectiles [9].

In the absence of highspeed photographic records of these processes, estimates of launch speeds have been based on spore capture on discs spinning at known angular velocity [10], [11] and from the interruption of light beams [12]. Other studies have relied on models that could only infer velocity from measured distances of discharge [13], [14]. However, typical spore launches involve initial velocities that are characterized by intermediate Reynolds numbers (Re≈10–100). Thus, the validity of these inferred estimates of launch speed is limited by the assumptions of the drag modeling. In this paper, we provide unambiguous measurements of launch speeds and acceleration using ultra-high-speed video recordings of discharge processes in four coprophilous fungi. These data allow the experimental evaluation of different drag models. In addition to these ballistic questions, there is considerable uncertainty about the magnitude of the pressures that power spore discharge and the identity of the compounds responsible for generating these pressures. Previous authors have suggested that enormous turgor pressures might be required to discharge spores, requiring the accumulation of very high concentrations of ions and sugars [2], [14]. We address this using a pair of complementary spectroscopic methods to provide an inventory of inorganic ions and sugars. The spectroscopic data show that spore discharge mechanisms are driven by very modest levels of pressure that are characteristic of the majority of fungal cells.

The fungi chosen for analysis were: *Ascobolus immersus*, *Podospora anserina*, *Pilobolus kleinii*, and *Basidiobolus ranarum*. *A. immersus* is a coprophilous ascomycete that discharges eight spores from each of its multiple asci that are exposed on the surface of a gelatinous fruiting body or ascoma. A second ascomycete, *P. anserina*, produces its asci within a flask-shaped ascoma or perithecium; its spores are harnessed to one another by means of mucilaginous appendages. The zygomycete *P. kleinii* produces a bulbous, fluid-filled stalk or sporangiophore, that squirts a spore-filled sporangium from the dung on which the fungus thrives. Finally, the zygomycete *B. ranarum* flourishes in the dung of amphibians and reptiles and causes rare infections in mammalian hosts, including humans [15]. Its spore-producing structure is reminiscent of the *Pilobolus* sporangium, but discharges a single spore, or conidium, rather than a sporangium.

## Results and Discussion

**Figure 1:**
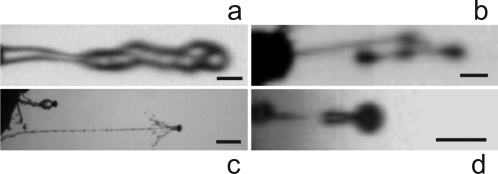
Single frames from high speed video recordings of spore discharge in four coprophilous fungi. a, *Ascobolus immersus*, with 8 ascospores discharged from ascus tip, 60 µs into the launch. b, *Podospora anserina*, with 4 ascospores harnessed by mucilaginous appendages, 96 µs after release from apex of fruiting body at left of frame. c, *Pilobolus kleinii*, sporangium with sap trailing behind, 0.8 ms after beginning of discharge. Undischarged sporangium at top of frame. d, *Basidiobolus ranarum*, single spore carrying portion of dehisced conidiophore, 24 µs into the launch. Scale bars, a, b, d, 50 µm, c, 1 mm. Frame rates a, 100,000 fps, b, 250,000 fps, c, 50,000 fps, d, 210,000 fps.

High speed videos reveal that the octet of spores of *A. immersus* is propelled as a single mass, embedded in mucilage and fluid from the ascus, so the drag upon the projectile is not determined by the shape of the individual spores (Fig. 1a, Video S1). In some cases, the spores remain in an elongated form during flight; in others, surface tension pulls the spores together after launch, forming a spherical projectile. The ballistics of *P. anserina* ascospores are similar, with the spores moving as an irregularly-shaped projectile held together by appendages and embedded in sap squirted from the ascus (Fig. 1b, Video S2). In *P. kleinii* the sporangium is propelled from the tip of the sporangiophore by a stream of ejected fluid (Fig. 1c, Video S3). The spore of *B. ranarum* is launched when the wall of the subtending conidiophore ruptures around its circumference, discharging its tip with the spore (Fig. 1d, Video S4). In half of the video sequences obtained from this species, the conical conidiophore tip separates from the spore during flight. Median launch speeds in the four species varied from 4 m s^−1^ (in *B. ranarum*) to 21 m s^−1^ (in *P. anserina*), with a maximum measured acceleration of 1.8×10^6^ m s^−2^ in *A. immersus* (Table 1). In terms of acceleration, these are the fastest recorded flights in nature.

**Table 1:** Ballistics of spore and sporangium discharge in four coprophilous fungi based upon high speed video analyses and different models for the effects of viscous drag on particle flight.

	*Ascobolus immersus*	*Podospora anserina*	*Pilobolus kleinii*	*Basidiobolus ranarum*
Measured launch speed (range, median, sample size)	5–18, 14 (12)	10–25, 21 (17)	2–13, 9 (14)	2–9, 4 (10)
Measured maximum acceleration (m s^−2^)	1,800,000	1,500,000	210,000	1,500,000
Measured maximum range (m)*	0.3	0.2	2.5	0.02
Estimated maximum range (m) using Stokes drag	0.3	0.2	2.9	0.05
Measured turgor pressure (MPa)	0.30 (strain gauge[13])	0.40 (spectroscopy)	0.55 (osmometry [19])	–
	0.40 (spectroscopy)		0.55 (spectroscopy)	
Estimated pressure (MPa)**	0.30–1.00	0.11–0.29	0.03–0.17	0.01–0.72

* Maximum range measurements by authors with exception of *P. klenii* data published by Buller [19].

** Pressure estimates (for measured range of launch speeds) were obtained by calculating the force needed to cause the observed projectile accelerations via Newton's second law and the area over which that force was applied.

There have been previous estimates of very high accelerations of fungal spores, but these were based on drag models that appear to be unsuited for the speeds characteristic of spores. Trail et al. [14] estimated initial accelerations of 8.5×10^6^ m s^−2^ during ascospore discharge in the wheat pathogen *Gibberella zeae*. This estimate was derived from a semi-empirical equation for the drag coefficient [16], the dimensions of the discharged spores, and an ascus range of 9 mm. The new video data from our study provide a unique opportunity to test different models for the effect of viscous drag on the motion of microscopic projectiles. Although the fast movement of the spores falls beyond the regime where Stokes Law is known to apply [16], this model nonetheless correctly predicts the measured ranges from the speeds determined from our video recordings (Table 1). The more complex drag model [14] underestimates these ranges by a factor of two or more (e.g., only 0.7 m for *P. klenii*). In order to reproduce the observed ranges, this model would require launch speeds and pressures an order of magnitude larger than those measured (e.g., 180 m s^−1^ and a non-physiological pressure of 2.3 MPa for *P. kleinii*).

A possible explanation for this discrepancy is that while the generally accepted correlation between drag coefficient and Reynolds number was experimentally determined via constant-velocity sedimentation studies [17], fungal spores show exceedingly rapid deceleration after launch. In a computational fluid dynamics paper, Wakaba and Balachandar showed that decelerating spheres are overtaken by a wake created in the surrounding medium [18]. Under these conditions, the moving object behaves as if it had additional mass (added mass force) and experiences less drag than a sphere moving at constant velocity. Initial calculations suggest that this effect accounts for a small proportion of the apparent reduction in drag in our study. It is clear that the non-equilibrium conditions experienced by decelerating spores complicates the drag modeling.

**Figure 2:**
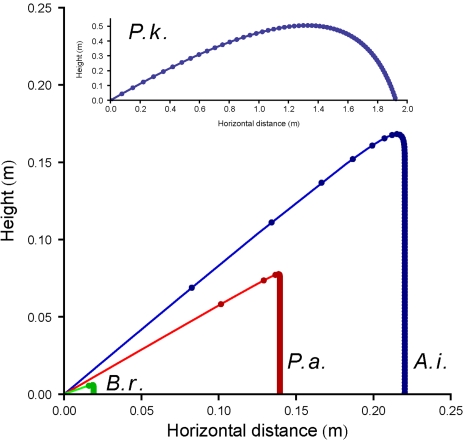
Predicted trajectories of spores and sporangia of four fungi based on launch data obtained by high speed video microscopy. Trajectories of spores and sporangia of *Ascobolus immersus* (*A.i.*, blue), *Podospora anserina* (*P.a.*, red), *Basidiobolus ranarum* (*B.r.*, green), and *Pilobolus kleinii* (*P.k.*, blue in inset). Points indicate projectile positions at 10 ms intervals. The truncated trajectories of *A. immersus*, *P. anserina*, and *B. ranarum* are indicative of the dominance of viscous forces over inertial forces in the motional regimes for these launches. Inertia is more significant for the flight of the larger sporangia of *P. kleinii*. Launch angles of 40° (*A.i.*), 30° (*P.a., P.k.*), and 20° (*B.r.*), were chosen to separate the trajectories from one another but also reflect the phototropic orientation of these asci, sporangiophores, and conidiophores in nature.

The agreement between the discharge distances predicted from our velocity data using Stokes drag and the measured ranges is remarkably good, given that the video recordings are limited to the initial launch events (Fig. 2). In the case of *P. klenii*, for example, the image in Fig. 1c shows the sporangium 0.8 ms after separation from its sporangiophore when it has traveled 8 mm, which is less than 1% of the maximum range of this species.

To enrich our picture of the discharge processes in these fungi, we used quantitative spectroscopic methods to determine the chemical composition of the ascus sap in *A. immersus* and *P. anserina*, and the sporangiophore sap in *P. kleinii*. The minuscule quantity of fluid ejected with the discharged spores of *B. ranarum* precluded chemical analysis of this species. In the three species examined, hydrostatic pressure was generated by the combined osmolality of sugar alcohols and inorganic ions. The dominant sugars were mannitol, glycerol, erythritol, and pinitol, but the relative concentrations varied between species. In the ascus sap of *A. immersus*, the combined concentration of mannitol and glycerol was 80 mM, and potassium and counter ions boosted the osmolyte concentration by 100 mM. The combined osmolality of these compounds will generate a turgor pressure of 0.44 MPa or 4.4 atm at maximum ascus hydration, which is consistent with a published measurement of the mean ascus turgor pressure of 0.31 MPa using a miniature strain gauge device [13]. The most abundant sugars in *P. anserina* were erythritol and pinitol, accounting for a combined concentration of 70 mM, which is very similar to the sugar content of the asci of *A. immersus*. In *P. kleinii*, the dominant sugars were mannitol, pinitol, and erythritol, but in this species, ions accounted for 95% of the total osmolality in the clear sporangiophore fluid. The corresponding turgor pressure estimate from the spectroscopic data was 0.50 MPa, which is in excellent agreement with published data [19]. Finally, published pressure measurements and estimates from spectroscopic data for *A. immersus*, *P. anserina*, and *P. kleinii* were consistent with the necessary pressures for the various launch mechanisms predicted from the simple Stokes model for drag (Table 1).

In this paper we have documented a series of remarkable feats of natural engineering, based on universal aspects of fungal structure and metabolism. The turgor pressures of <1.0 MPa (10 atm) that power these supremely fast movements are no higher than those measured from fungal hyphae [20], suggesting that explosive mechanisms of spore discharge do not require any extraordinary mechanisms of osmolyte accumulation, nor the elaboration of any specialized cell wall structures to maintain this pressure prior to discharge. Unusual features of these mechanisms include the controlled and rapid rupture of the pressurized squirt guns that allow the nearly instantaneous release of energy and discharge of the spores and sporangia. The match between predicted and measured flights also suggest that very little of this energy is lost to friction during the earliest phases of spore release. The launch speeds of the species in this study are likely to be among the fastest among any fungi because their coprophilous ecology has demanded much longer ranges than those necessary for the dispersal of species that need only escape boundary layers.

## Materials and Methods

### Culture methods

*Ascobolus immersus* strains 18558 (+) and 18559 (−) obtained from the American Type Culture Collection (ATCC, Manassas, VA), were crossed on horse dung agar [13], incubated at room temperature in the dark until ascomata developed, then exposed to continuous illumination during the experiments. *Podospora anserina* strains F7300 (S^−^) and F7301 (S^+^), kindly supplied by A. Hamann (University of Frankfurt), were treated in the same way. *Pilobolus kleinii* strain 14499 (ATCC) was cultured on rabbit dung agar, incubated in the dark for 5–7 d, then exposed to 12 h dark/12 h light to induce sporangiophore formation. *Basidiobolus ranarum* strain Br02 was isolated from frog dung and grown on Czapek-Dox agar under 12 h dark/12 h light to induce conidiophore formation.

### Ultra-high speed video microscopy

Video recordings were made with FASTCAM-ultima APX and APX-RS cameras (Photron, San Diego, CA) attached to a binocular dissecting microscope and to an inverted compound microscope fitted with long-working distance objectives (Olympus, Tokyo). Each video clip was compiled from ≤100 image files extracted from recordings consisting up to 1 million images captured in ≤4 s (e.g., 1 million image files captured with 2 µs shutter at 250,000 fps in 4 s). Analysis of digital images was performed with VideoPoint v.2.5 (Lenox Softworks, Lenox MA), Image-Pro Plus 6.2 (Media Cybernetics, Bethesda, MD), and proprietary software from Photron.

### Measuring ascus range

Discharge distances were measured by attaching culture plates in a vertical orientation at one end of an acrylic box (42×42×6 cm) using Velcro® tape. The inner walls of the box were covered with black paper to exclude light, with the exception of a 5×5 cm widow cut into the far end of the box through which a light source was directed. Wet paper towels were placed in the box to maintain high humidity. Numbered microscope slides were placed in straight paths beneath the culture plates to catch spores after horizontal discharge from their phototropic asci [11]. Spores on each slide were counted to produce spore density versus distance plots.

### Analysis of sap composition

Sap expelled from asci and sporangiophores was collected on the underside of Petri dish lids above sporulating cultures. The lids were air dried and the number of spores captured on each lid was counted under a dissecting microscope. Measurements of the mean sap volume shot from the asci or sporangiophores of each species were made from light microscopic images of mature asci; these values were multiplied by the number of spore clusters (12.5% of total spore count in *A. immersus*, 25% of spore count in *P. anserina*) or sporangia (of *P. kleinii*) to provide estimates of sap volume deposited on each Petri dish lid. Water soluble ions and organic compounds in the sap were harvested by swirling 1 mL of sterile distilled water in the inverted lids. Aqueous extracts were then transferred to microfuge tubes. Most of the spores remained attached to the lids, but spores that were transferred with the aqueous extracts were removed by centrifuging at 10,000 *g* for 5 min. The supernatants from the microfuge tubes were then stored at –20°C. Sugars and sugar alcohols contributing to sap osmolality were identified and quantified using GC/MS. Samples were derivatized to produce alditol acetates of the sugar alcohols [21]. The dried samples were resuspended in 10 µL of chloroform and multiple injections of 1 µL, separated by blank runs of chloroform, were analyzed on a Varian CP-3800 GC/Saturn 2000 MS. Osmolytes were identified by comparison to GC/MS of purified samples of alditol acetates, and mass spectra of alditol acetates obtained from the NIST/EPA/NIH Mass Spectral Library (NIST, Gaithersburg, MD). The concentrations of the major osmolytes were determined from standard curves produced by plotting the log of the concentration of standards versus the log of the ion intensity of a characteristic ion fragment for each osmolyte (103 for glycerol, and 139 for mannitol). Metals analysis was carried out using a Varian 800 series ICP-MS (Mulgrave, Victoria, Australia) controlled with Varian ICP-MS Expert software. Samples harvested from Petri dish lids were diluted to 10 mL and ion concentrations were determined from standard curves produced from dilutions of ion standards in nitric acid (Inorganic Ventures, Inc., Lakewood, NJ).

### Mathematical model

Spore flight trajectories were modeled in two ways. Stokes' law describes the drag force on a spherical particle moving through a viscous fluid in the laminar flow, low Reynolds number (Re<1) regime in which viscous forces dominate over inertial forces. In this model, 
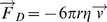
 where 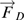 is the force due to viscous drag, *r* is the aerodynamic radius of the projectile, *η* is the viscosity of the air, and 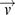 is the projectile velocity. This vector force, combined with Newton's second law, can be analytically integrated to yield expressions for the *x*- and *y*-positions of the spore as functions of time which can be plotted parametrically to determine the spore trajectory. An analytical expression for the range of the projectile can also be derived.

Spore trajectories were also calculated using a more complicated, quasi-empirical model for the drag which has been proposed for particles moving through fluids at the onset of turbulence, a regime characterized by Reynolds numbers between 1 and 1,000 [16]. In this model,
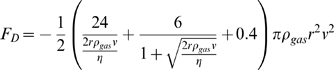
where *ρ_gas_* is the viscosity of the air. This expression can be combined with Newton's second law but cannot be integrated analytically. Instead, it must be numerically integrated using any standard numerical integration algorithm. In some cases, acceleration was computed from the position of the projectiles in multiple video frames, but in others, the accelerations were accomplished so swiftly that we estimated acceleration from the observed change in velocity during the time interval between two successive frames.

## Supporting Information

Video S1 Ascobolus immersus, 1000,000 fps.
(0.33 MB AVI)

Video S2 Podospora anserina, 250,000 fps.
(0.33 MB AVI)

Video S3 Pilobolus kleinii, 50,000 fps.
(4.92 MB AVI)

Video S4 Basidiobolus ranarum, 210,000 fps.
(0.19 MB MPG)
